# On the relationship between serial interval, infectiousness profile and generation time

**DOI:** 10.1098/rsif.2020.0756

**Published:** 2021-01-06

**Authors:** Sonja Lehtinen, Peter Ashcroft, Sebastian Bonhoeffer

**Affiliations:** Institute for Integrative Biology, Department of Environmental System Science, ETH Zürich, Zürich, Switzerland

**Keywords:** epidemiology, SARS-CoV-2, generation time, contact tracing, modelling, infectiousness

## Abstract

The timing of transmission plays a key role in the dynamics and controllability of an epidemic. However, observing generation times—the time interval between the infection of an infector and an infectee in a transmission pair—requires data on infection times, which are generally unknown. The timing of symptom onset is more easily observed; generation times are therefore often estimated based on serial intervals—the time interval between symptom onset of an infector and an infectee. This estimation follows one of two approaches: (i) approximating the generation time distribution by the serial interval distribution or (ii) deriving the generation time distribution from the serial interval and incubation period—the time interval between infection and symptom onset in a single individual—distributions. These two approaches make different—and not always explicitly stated—assumptions about the relationship between infectiousness and symptoms, resulting in different generation time distributions with the same mean but unequal variances. Here, we clarify the assumptions that each approach makes and show that neither set of assumptions is plausible for most pathogens. However, the variances of the generation time distribution derived under each assumption can reasonably be considered as upper (approximation with serial interval) and lower (derivation from serial interval) bounds. Thus, we suggest a pragmatic solution is to use both approaches and treat these as edge cases in downstream analysis. We discuss the impact of the variance of the generation time distribution on the controllability of an epidemic through strategies based on contact tracing, and we show that underestimating this variance is likely to overestimate controllability.

## 1. Background

### 1.1. Motivation

Estimating the generation time (the timing between successive infections in a transmission chain) distribution in an emerging epidemic is both extremely important and extremely challenging. Generation time is key to assessing the controllability of the epidemic: it determines the relationship between the basic reproductive number *R*_0_ and the epidemic’s growth rate [1,2], as well as how much delays in the isolation of infected individuals impede epidemic control [3,4]. However, the timing of transmission events is often unknown. The distribution of generation times is therefore typically estimated based on the timing of symptom onset, which requires assumptions about the relationship between infectiousness and symptoms. These assumptions are not always explicitly stated and their plausibility is rarely discussed. Here, we illustrate how assumptions about infectiousness and symptom onset affect the relationship between the generation time and serial interval distributions, and the implications this has for assessing epidemic controllability.

### 1.2. Definitions

**Figure 1.**
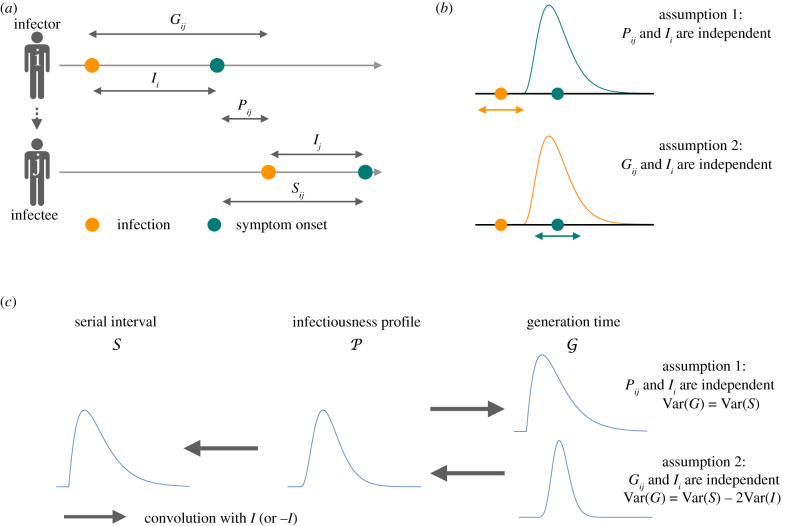
A schematic of how the assumptions about infectiousness and symptoms affect the relationship between the serial interval and generation time distributions. (*a*) Definitions of: serial interval *S*_*ij*_, time from symptom onset of infector *i* to symptom onset of infectee *j*; generation time *G*_*ij*_, time from infection of *i* to infection of *j*; incubation time *I*_*i*_, time from infection of *i* to symptom onset of *i*; and *P*_*ij*_, time from symptom onset of *i* to infection of *j*. (*b*) Illustration of how infectiousness relates to the point of infection and onset of symptoms under the two different assumptions. Under assumption 1 (*P*_*ij*_ and *I*_*i*_ independent), the infectiousness is fixed with reference to symptom onset. Under assumption 2 (*G*_*ij*_ and *I*_*i*_ independent), the infectiousness is fixed with reference to the point of infection. (*c*) The relationship between the generation time distribution, the infectiousness profile and the serial interval distribution under assumptions 1 and 2.

We consider an infector *i* and infectee *j* (figure 1*a*) and define: *S*_*ij*_ as the serial interval (time interval between symptom onset of infector *i* and symptom onset of infectee *j*); *G*_*ij*_ as the generation time (time interval from infection of *i* to infection of *j*); *P*_*ij*_ as the time interval from symptom onset of *i* to infection of *j*; and *I*_*i*_ as the incubation period of *i* (and *I*_*j*_ is the incubation period of *j*). For clarity, we drop the indices when they are not necessary. We use calligraphic letters to denote the probability density functions—i.e. distributions—of these time variables (e.g. S is the serial interval distribution). The generation time distribution G describes infectiousness relative to the point of infection, while P describes infectiousness relative to symptom onset. We refer to P as the infectiousness profile [5,6].

### 1.3. Estimation based on transmission pairs

The distributions I, G, P and S are typically derived from contact tracing data during epidemic outbreaks. Such data consist of transmission pairs, usually with symptom onset times for infector and infectee, and an exposure window for the infection time of the infectee. These data allow I and S to be estimated without further assumptions, but not P and G.

Here, we note some general caveats relating to the use of transmission pairs to estimate these distributions. These are not relevant to the relationship between G and S discussed here, but should nevertheless be considered when working with these data. Firstly, in a growing epidemic, contact tracing data will underestimate generation times and serial intervals: when prevalence is increasing, sampled cases will be biased towards recent infections. This bias can be corrected by explicitly accounting for the growth when deriving the distributions [2], as done, for example, in Ferretti *et al.* [4]. Secondly, as prevalence increases and the number of susceptible individuals becomes limiting, generation times and serial intervals will contract: each susceptible can only be infected once, resulting in fewer longer intervals [7]. Thirdly, sampled transmission pairs may not be representative of the overall population—for example, asymptomatic cases will be under-represented. Furthermore, contacts who are exposed to infection but not infected contribute information about infectiousness that is not captured in these analyses.

## 2. Relationship between G, P and S

### 2.1. Deriving G and P from S

The relationships between the time intervals *G*_*ij*_, *P*_*ij*_ and *S*_*ij*_ are illustrated in figure 1*a* and are captured by the following equations:

2.1a $$S_{ij}=P_{ij}+I_{\;j},$$

2.1b $$G_{ij}=P_{ij}+I_{i}$$

2.1c $$\text{and}\;\;G_{ij}=S_{ij}+I_{i}-I_{\;j}.$$

Deriving P from S does not require strong assumptions; *P*_*ij*_ and *I*_*j*_ are plausibly independent: it is reasonable to assume that the interval between the infector’s symptom onset and onward transmission does not affect the incubation period of the infectee. From equation (2.1*a*), we can then write S as the convolution between P and I, i.e.

2.2 S=P ∗ I.

The infectiousness profile P can therefore be derived by deconvolution of the serial interval and incubation period distributions [5,6].

Deriving G is not as straightforward: as the intervals *P*_*ij*_ and *I*_*i*_ relate to the same individual, independence of the two is a more debatable assumption than for *P*_*ij*_ and *I*_*j*_. Progress can be made by assuming that *I*_*i*_ and *I*_*j*_ are independent and identically distributed (i.i.d): under this assumption, the intervals *S* and *G* have the same mean and their variances are related by the covariance of *P*_*ij*_ and *I*_*i*_ (from equations (2.1*a*,*b*)) [8]

2.3 $$\text{Var}(G)=\text{Var}(S)+2\text{Cov}(P_{ij},I_{i}).$$

Deriving the generation time distribution G requires further assumptions: typically, either the independence of *P*_*ij*_ and *I*_*i*_ or the independence of *G*_*ij*_ and *I*_*i*_.

### 2.2. Assumption 1: independence of incubation period of the infector (*I*_*i*_) and time from symptoms of infector to infection of infectee (*P*_*ij*_)

Under this assumption, infectiousness is fixed with reference to symptom onset (figure 1*b*): there is no correlation between how long it takes an individual to develop symptoms and the interval between symptom onset and onward transmission. Such a situation would arise, for example, if individuals have a variable period between infection and the onset of infectiousness, the duration of which does not affect subsequent infectiousness or onset of symptoms (see also [2]).

Using equation (2.1*b*), the independence of *P*_*ij*_ and *I*_*i*_ means that G can be derived as the convolution of P and I (i.e. G=P ∗ I) and is thus identical to S (equation (2.2)). Thus, the often-used approach of approximating the generation time distribution by the serial interval implicitly makes this assumption (figure 1*c*).

In line with the above, under this assumption, the variance of *G* is equal to the variance of *S*,

2.4 $$\begin{array}{rl} \text{Var}(G) & =\text{Var}(S)+2\text{Cov}(P_{ij},I_{i})\\ & =\text{Var}(S). \end{array}$$

This assumption is biologically implausible: it requires the incubation period to be independent of processes affecting infectiousness. Yet infectiousness and symptom onset are both likely to depend on pathogen load; it is therefore unlikely that assumption 1 holds for most pathogens. Furthermore, unlike serial intervals, generation times cannot be negative. When observed, negative serial intervals are empirical evidence against assumption 1.

### 2.3. Assumption 2: independence of incubation period of the infector (*I*_*i*_) and time from infection of infector to infection of infectee (*G*_*ij*_)

Under this assumption, infectiousness is fixed with reference to the point of infection (figure 1*b*): the timing of transmission is uncorrelated with the timing of symptom onset. As *P*_*ij*_ = *G*_*ij*_ − *I*_*i*_ (equation (2.1*b*)), P would then be the convolution of G and −I, P=G∗(−I). The generation time distribution *G* could therefore be derived from *S* by deconvolving first with I and then with −I (figure 1*c*), i.e. solving S=G ∗ (−I) ∗ I for G. The functional form of G would therefore depend on both empirical distributions S and I. This is the approach adopted for deriving the generation interval of severe acute respiratory syndrome–coronavirus 2 (SARS-CoV-2) in Ferretti *et al.* [4] and Ganyani *et al.* [9].

In line with the above, under this assumption, the variance of *G* is smaller than the variance of *S*,

2.5 $$\begin{array}{rl} \text{Var}(G) & =\text{Var}(S)+2\text{Cov}(P_{ij},I_{i})\\ & =\text{Var}(S)+2\text{Cov}(G_{ij}-I_{i},I_{i})\\ & =\text{Var}(S)+2[\text{Cov}(G_{ij},I_{i})-\text{Cov}(I_{i},I_{i})]\\ & =\text{Var}(S)-2\text{Var}(I)\\ & \leq \text{Var}(S). \end{array}$$

This assumption is also biologically implausible. If infectiousness and symptom onset both depend on pathogen load, individuals with a rapid increase in pathogen load will develop symptoms early (short *I*_*i*_) and transmit sooner after infection (small *G*_*ij*_), leading to Cov(*G*_*ij*_, *I*_*i*_) > 0. Furthermore, symptom onset itself is likely to affect infectiousness. Depending on the pathogen, the effect could be in either direction (symptomatic individuals transmitting more because symptoms contribute to transmission, or symptomatic individuals transmitting less because they self-isolate). However, either scenario would lead to a positive correlation between the timing of symptom onset and transmission (electronic supplementary material, figure S1).

### 2.4. Assumptions 1 and 2 bound the variance of *G*

Although neither assumption 1 nor assumption 2 is plausible, they are still informative: the variances of the generation time distribution derived under these assumptions can reasonably be considered as upper and lower bounds for Var(*G*),

2.6 Var(S)−2Var(I)≤Var(G)≤Var(S).

Assumption 1 leads to the upper bound Var(*G*) = Var(*S*). A greater variance would require Cov(*P*_*ij*_, *I*_*i*_) > 0 (see equation (2.3)), i.e. transmission occurring late with reference to symptoms for individuals with a longer incubation period—for example, a greater proportion of transmission being post-symptomatic when symptoms appear late. The notion that Cov(*P*_*ij*_, *I*_*i*_) > 0 is unlikely has also been previously suggested in the literature [2]. Furthermore, if negative serial intervals are observed, this suggests Var(*G*) < Var(*S*) (assuming that the serial interval distribution and generation time distribution have a similar shape), since the distributions have the same mean and negative generation times are not possible.

Assumption 2 leads to the lower bound Var(*G*) = Var(*S*) − 2Var(*I*). A lower variance would require Cov(*G*_*ij*_, *I*_*i*_) < 0 (see equation (2.5)), i.e. transmission occurring soon after infection for individuals with a longer incubation period. However, as discussed above, individuals with a faster increase in pathogen load are likely to start transmitting earlier and also have a shorter incubation period, leading to Cov(*G*_*ij*_, *I*_*i*_) > 0. Furthermore, if the appearance of symptoms leads to a change in infectiousness (in either direction), earlier symptoms will correlate with earlier transmission, again leading to Cov(*G*_*ij*_, *I*_*i*_) > 0.

## 3. Possible solutions

### 3.1. Empirical testing of assumptions

*A priori*, there is no reason to consider either assumption 1 or assumption 2 as more plausible than the other. With appropriate data, the assumptions can be tested empirically. For example, such analysis for SARS-CoV-2 suggests a strong positive correlation between *G*_*ij*_ and *I*_*i*_, and a weak negative correlation between *P*_*ij*_ and *I*_*i*_ [10]. In other words, for SARS-CoV-2, neither assumption holds, but assumption 1 (independence of *P*_*ij*_ and *I*_*i*_) is a better approximation.

The empirical testing of these assumptions requires transmission pairs for which *I*_*i*_ and *G*_*ij*_ (or, equivalently, *P*_*ij*_) can be estimated. This can be done with either: (i) data on the infection time for both infector *i* and infectee *j* and the symptom onset time for *i* or (ii) data on the symptom onset time for both *i* and *j* and the infection time for *i*, as the assumption that *P*_*ij*_ and *I*_*j*_ are independent allows the infection time for *j* to be estimated. Therefore, an interesting corollary here is that, for transmission pairs with a known serial interval, data on the infection time of the infector is more informative than the infection time of the infectee.

In practice, when such data are available, the generation interval distribution can simply be directly estimated from the data [10]. The reason for deriving G from S is precisely the lack of such data; an alternative approach for assessing the plausibility of the assumptions underlying this derivation is therefore necessary.

### 3.2. Assumptions 1 and 2 as edge cases

As assumptions 1 and 2 bound the variance of *G*, a solution when data are lacking is to derive G under both assumptions, and treat these as boundary cases in downstream analysis (e.g. best and worst case scenarios). This approach may not always be entirely straightforward. If Var(*S*) < 2Var(*I*), assumption 2 would lead to negative variance of *G*. In these cases, the lower bound for Var(*G*) is zero. If the serial interval distribution includes negative values, deriving G under assumption 1 is problematic. A pragmatic approach in these cases would be to use Var(*G*) = Var(*S*) and to assume a non-negative functional form for G (e.g. lognormal, gamma or Weibull), although the resulting distribution will not be the correct distribution under assumption 1. The key point is that evidence against assumption 1, such as negative serial intervals, is not, in itself, evidence in favour of assumption 2.

## 4. Implications for the modelling of contact tracing

Finally, we explore the impact of the variance and functional form of the generation time distribution on the modelling of contact tracing, using the example of SARS-CoV-2. Table 1 shows empirical estimates for the mean and standard deviation (s.d.) of the serial interval and incubation period. Both have a mean of around 5 days. The s.d. of the incubation period is generally estimated to be in the range of 2.3–2.8 days, although some studies have also reported considerably higher values (table 1). With the exception of some smaller studies, the s.d. of the serial interval is generally estimated to be of the order of 4.2–5.5 days. Assuming the s.d. of *S* to be 5 days [Var(*S*) = 25] and the s.d. of *I* to be 2.8 days [Var(*I*) = 8], a plausible range for the s.d. of *G* would thus be 5 to 3 days [Var(*G*) = 25 and Var(*G*) = 9)] under assumptions 1 and 2 respectively—though lower values cannot be excluded if the lower estimates of the s.d. of *S* or higher estimates of the s.d. of *I* hold.

**Table 1.** Shape, mean and variance of incubation period and serial interval distributions of SARS-CoV-2 from a range of studies. *N* indicates the sample size.

study	distribution	shape	mean (days)	standard deviation (days)	*N*
Zhang *et al.* [11]	incubation	lognormal	5.2	2.6	49
Li *et al*. [12]	incubation	lognormal	5.2	3.9	10
Lauer *et al*. [13]	incubation	lognormal	5.5	2.4	181
Backer *et al*. [14]	incubation	Weibull	6.4	2.3	88
Linton *et al*. [15]	incubation	lognormal	5.6	2.8	158
Ganyani *et al*. [9]	serial interval (Singapore)	gamma	5.2	4.3	54
Ganyani *et al*. [9]	serial interval (Tianjin)	gamma	4.0	4.2	114
Zhang *et al*. [11]	serial interval	gamma	5.1	2.7	34
Li *et al*. [12]	serial interval	gamma	7.5	3.4	6
He *et al*. [5]	serial interval	gamma (shifted)	5.8	4.5	77
Nishiura *et al*. [16]	serial interval	lognormal	4.7	2.9	28
Ali *et al*. [17]	serial interval (all)	normal	5.1	5.3	677
Ali *et al*. [17]	serial interval (pre-peak)	normal	7.8	5.2	162

**Figure 2.**
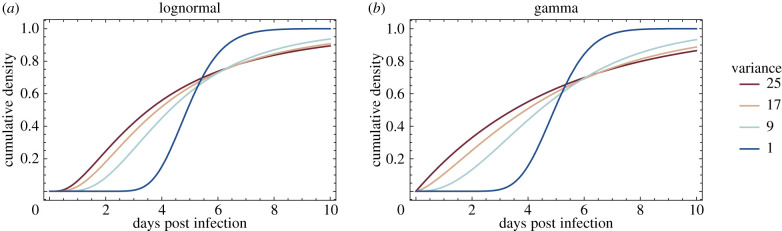
A schematic showing the impact of functional form and variance on the timing of onward transmission. The plots show cumulative generation time distributions, i.e. the proportion of transmission occurring within *x* days of infection. All distributions have a mean of 5 days. The illustrated variances correspond to standard deviations of 5.0, 4.1, 3 and 1 days. Note that lognormal (*a*) and gamma (*b*) distributions have support on (0, ∞), implying an infinite infectious period, which is not correct. However, in practice, this is an acceptable approximation when the probability density in the tail of the distribution is very low.

Figure 2 illustrates how the variance and functional form of the generation time distribution impact how quickly infected individuals need to be isolated to prevent a significant portion of onward transmission, that is, how quickly contact tracing needs to operate for the epidemic to be controllable. For example, assuming that the generation time is gamma distributed with a mean of 5 days, preventing 80% of onward transmission requires isolation of an infected individual within 1.1 days if the s.d. of *G* is 5 days, and 2.5 days if the s.d. of *G* is 3 days. On the other hand, if the variance is large, isolating individuals even with considerable delay will still have an impact on onward transmission. For example, isolating an infected individual 10 days after infection will prevent 14% of onward transmission if the s.d. of *G* is 5 days, but only 7% if the s.d. of *G* is 3 days. In practice, if the goal of contact tracing is to control the epidemic, the former scenario is more relevant [4]. Thus underestimating the variance of the generation time distribution (assumption 2) risks overestimating the effectiveness of contact tracing.

## 5. Conclusion

Neither of these two commonly used approaches for estimating the generation time distribution from the serial interval distribution is based on plausible assumptions for most pathogens. The two approaches yield generation time distributions with the same mean, but different variances. This difference in variance can have a considerable impact on estimating the controllability of an epidemic through contact tracing. The two variances are plausible upper and lower bounds for the variance of the generation time distribution. We therefore suggest that a pragmatic solution is to treat the distributions derived through the two approaches as edge cases in downstream analysis. When implementing this solution, it remains important to correct for the bias towards short intervals arising in a growing epidemic and to consider the limitations of analyses based on contact tracing data.

## Supplementary Material

Figure S1

## Data accessibility

All data are available within the article.
