# Supplementary material for: On the relationship between serial interval, infectiousness profile and generation time
Source: J R Soc Interface. 2021 Jan 6;18(174):20200756. doi: 10.1098/rsif.2020.0756 (PMC7879757; doi:10.1098/rsif.2020.0756)
Supplement: Figure S1 [file rsif20200756supp1.pdf]

Supporting Figure

Sonja Lehtinen, Peter Ashcroft & Sebastian Bonhoeffer
0n the relationship between serial interval, infectiousness profile and generation time
Journal of the Royal Society Interface

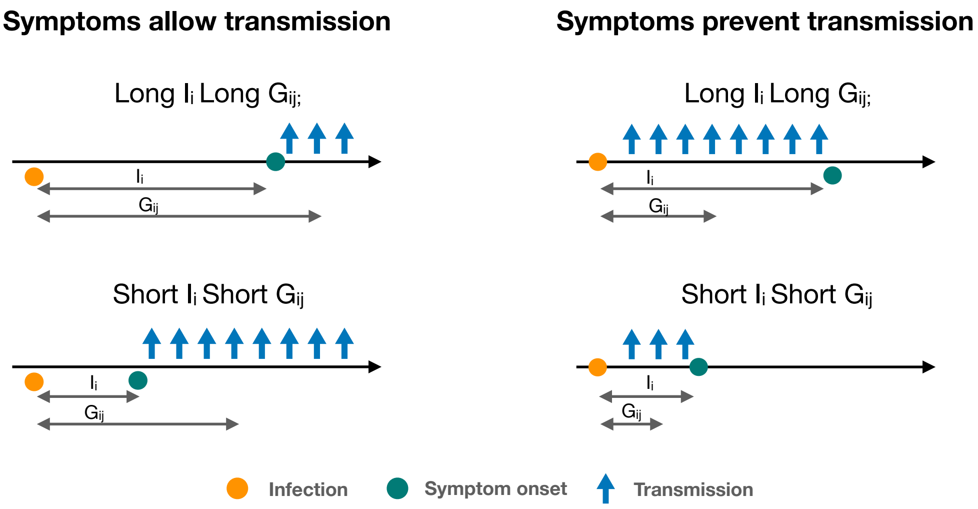

Figure 1: Schematic illustrating how symptom onset affecting transmission leads to a positive correlation between incubation period and generation time, whether symptoms increase or decrease transmission. In the left-hand panels, transmission only occurs after symptom onset. In the right-hand panels, transmission only occurs prior to symptom onset. We make the additional assumption that the length of the incubation period does not affect when infectiousness ends (for the left-hand panels) or starts (for the right-hand panel).
